# The Role of School-Related Well-Being for Adolescent Subjective Health Complaints

**DOI:** 10.3390/ijerph16091577

**Published:** 2019-05-06

**Authors:** Tomas Vaičiūnas, Kastytis Šmigelskas

**Affiliations:** 1 Health Research Institute, Faculty of Public Health, Medical Academy, Lithuanian University of Health Sciences, LT-47181 Kaunas, Lithuania; 2 Department of Health Psychology, Faculty of Public Health, Medical Academy, Lithuanian University of Health Sciences, LT-47181 Kaunas, Lithuania; kastytis.smigelskas@lsmuni.lt

**Keywords:** adolescent, health, somatic complaints, school, well-being

## Abstract

Background: This study aimed to explore the prevalence of chronic specific-site and multisite pain in adolescents and to investigate how it can possibly be determined by school-related factors. Methods: A population-based cross-sectional study was conducted in 2014 in Lithuania as a Health Behavior in School-Aged Children (HBSC) survey. The sample consisted of 5730 school children, aged 11, 13, and 15 years. The analyzed data focused on the school-related context (relations with family, peers, and teachers; school demand, satisfaction, and bullying) of adolescents and subjective health complaints. The relationships between social support and health complaint variables were estimated using multivariate analyses. Results: The most common subjective health complaint among respondents was a headache. Backache, headache, and stomachache were more common among girls than boys. All somatic complaints were expressed more in younger ages. Multisite complaints were more common among girls and were associated with age—older ones reported more complaints. School-related bullying, school demand, satisfaction, and social support were the most relevant and independent factors for multisite somatic complaints among adolescents.

## 1. Introduction

Adolescence is quite a sensitive development stage for managing resources, mental health, and health-related habits. For most young people, this is a healthy and happy experience, but for some it is emphasized by many social and health challenges [1,2,3]. There is a general agreement in the literature that healthy adolescent development has its roots in multiple contexts.

Somatic complaints, especially headache, stomachache, backache, and morning fatigue are frequently reported among children and adolescents and often co-occur [1,4,5,6,7,8]. Furthermore, multisite but not specific-site somatic complaints are more common among youth [9,10,11,12,13] and pain symptoms become more prevalent with age [9,10,11]. In many cases, it associates with behavioral [14,15], somatic [16,17,18], sociodemographic [11,16,17,19], and social [14] factors.

Predominantly, the research shows that youth who are involved in contexts that provide positive resources from important people (for example, parents, schools, peers or communities), are not only less likely to experience negative outcomes but are more likely to show positive development and health-related behaviors as well [20,21,22,23,24]. In line with these empirical findings, poor social support seems to be one of the possible predictors of subjective health complaints. With all potentially relevant external factors, psychosocial dimensions—perceived social and mental support from parents, teachers, and peers—often act as essential conditions and are strongly associated with young people’s behavior and health outcomes [23,25,26,27,28]. In addition, social support may have both direct and indirect effects on health-related outcomes [29,30].

Active support, especially from relatives, may be regarded as one of the most consistent predictors of health complaints [29,30,31], while the teacher–student relations also associate with wellness [32]. However, while parents are the most central source of support for young children, relational orientation changes tend to occur in adolescence, where peers also become more important [33,34,35]. Studies show that peer support acts as a strong buffering resource, especially regarding health complaints, while the support from teachers and parents demonstrates moderating effects [33,35,36].

School-related adolescent well-being is strongly associated with perceived aggressive behaviors [23,37,38], which is quite prevalent in the school setting [20,39]. Victims of bullying have significantly higher chances of developing new psychosomatic problems [37,40]. Bullying is associated with several subjective health complaints, among them—headache, backache, abdominal pain and dizziness, fatigue, and sleep problems [5,41,42]. Perceived social support plays an essential role as a protective factor against bullying and health complaints at the same time—higher parental warmth and support is associated with less involvement across all forms and classifications of bullying among adolescents [43,44,45,46], while peers and friends also seem to play a very important though more mixed role [44,47]. In the context of school, peers and teachers are more likely to be an essential part of a child’s social support network, providing social support in its many forms [43,48].

The present study aimed (1) to describe and compare the prevalence of chronic specific-site (headache, backache, or stomach-ache) and multisite pain in adolescents; (2) to investigate the patterns of chronic pain by age and gender; (3) to examine effects on variations of chronic pain, and associations between pain and age; and (4) to explore how school-related context (relations with family, peers, and teachers; school demand, satisfaction and bullying) can be related to adolescents’ subjective health outcomes (specific-site and multisite pain).

## 2. Materials and Methods

### 2.1. Data Collection

A population-based cross-sectional study was conducted in 2013–2014 as a Health Behavior in School-Aged Children (HBSC) survey, which is a collaborative project of the World Health Organization Regional Office for Europe. The analyzed data focussed on school-related context (relations with family, peers, and teachers; school demand, satisfaction and bullying) of adolescents and subjective health complaints. Data were collected by school-based surveys, using a standard international study protocol [49]. In Lithuania, random sampling in a cohort of school children aged 11, 13, and 15 years (on average, according to the HBSC protocol, to cover 1500 or more students) was used, ensuring that the sample is representative of the age and gender range. In total, 129 schools with 356 classes from Lithuania were included. Questionnaire forms were distributed in school classrooms by researchers and one schooling period (45 min) were provided as the time frame for filling in the questionnaire. Eligible participants could freely choose to participate or not in the survey. Measures of anonymity and confidentiality were ensured. Data of Lithuanian records were revised by the HBSC Data Management Centre at the Department of Health Promotion and Development, University of Bergen, Norway and the final sample consisted of 5730 school children.

Ethics approval for the study was provided by the Kaunas Regional Biomedical Research Ethics Committee (reference number BE-2-16) and was in line with the local practice for school surveys. The study was also endorsed by regional educational authorities and the national ministry of education.

### 2.2. Sample

The analyzed sample consisted of 5730 school children in Lithuania. Detailed characteristics of the study sample by age, gender, and family affluence are presented in Table 1.

### 2.3. Instruments

School-related well-being was measured through assessing social support from school teachers, classmates, and family; school-related stress—through school satisfaction, school effort/demands, and bullying at school; subjective health complaints—through the HBSC somatic-related symptoms checklist (HBSC–SCL); and sociodemographic factors—family affluence, age, and gender.

#### 2.3.1. School-Related Well-Being

For evaluation of social support, the Teacher and Classmate Support Scale [50,51] was used. Teachers support was evaluated using three question items: “I feel that my teachers accept me as I am”, “I feel that my teachers care about me as a person”, and “I feel a lot of trust in my teachers”. Response options ranged from 1—“very strongly disagree” to 5—“strongly agree” for both teacher and classmate scales. Peer support was measured with classmates support scale, consisting of three items: “The students in my class(es) enjoy being together”, “Most of the students in my class(es) are kind and helpful”, and “Other students accept me as I am”. For analyses, the mean scale score for both teachers and classmates support scales was dichotomized—values from 1 to 2 pts were considered as “High support”, and a score higher than 2—as “Low support”. Confirmatory factor analysis from several European countries supported a two-factor structure for these support scales and confirmed the test-retest reliability and measurement invariance across HBSC countries [50,52].

Assessment of perceived family support was carried out using the Multidimensional Scale of Perceived Social Support (MSPSS) [53]. The MSPSS has been well-validated and used in multiple studies and across different cultural contexts [54,55,56]. Family support was evaluated by four items from the family subscale: “My family really tries to help me”, “I get the emotional help and support I need from my family”, “I can talk about my problems with my family”, and “My family is willing to help me make decisions”. Dichotomization of scale scores was defined using the scale response descriptors, where the mean scale scores higher than 5 pts were considered as “High support “, and scores lower than 5 as “Low support”. The internal consistency of the social support scales was 0.84 for teachers, 0.82 for peers, and 0.85 for family.

Due to correlations between different social support types, for the overall social support analysis the three scales composed one composite indicator—overall social support score. This score ranged from 0 to 3, indicating how many support types were high for every participant in the study. The sufficient social support was considered as 2 or 3 points of overall social support score.

#### 2.3.2. Subjective Health Complaints

Subjective health complaints (SHC) were measured through the Health Behavior in School-Aged Children Symptom Checklist (HBSC-SCL [50]), a short, self-administered screening instrument that showed the frequency of eight common health complaints among children. Adolescents were asked: “In the last six months how often have you had the following?” and the items included were—a headache, stomachache, backache, depressed mood, irritability, nervousness, sleeping difficulties, and dizziness. The scales internal consistency was 0.84. For this study, we chose three somatic-related symptoms—headache, stomachache, and backache. Each somatic-related complaint was rated on a five-point frequency scale from 1—“rarely or never” to 5—“about every day”. Specific-site pain was considered as present in one specific location regardless of other locations. The multisite pain was considered as manifesting in at least two sites of the three analyzed (head, stomach, back).

#### 2.3.3. School-Related Stress

School satisfaction was measured using the item “How do you feel about school at present?”. This question has been found to be a powerful correlate of health-related perceptions [7,8,57,58].

School effort/demands were measured through the item about the general feeling of being pressured by the demands of school work, which included work at school and homework. Adolescents were asked, “How pressured do you feel by the schoolwork you have to do?”. In previous studies, it was often considered as a measure of school-related stress, and associations have been documented with frequent health complaints [22].

For measurement of bullying perpetration, the adolescents were asked: “How often have you taken part in bullying another student(s) at school in the past couple of months?” This question was preceded by description of bullying “A student is being bullied or victimized when he or she is exposed, repeatedly and over time, to negative actions on the part of one or more other students” [59]; this has been well-used and validated in empirical studies in multiple countries [5,60], and is used in the HBSC protocol [4,49]. Reports of at least 2 or 3 times a month in the past couple of months have been considered as a chronic bullying [61,62].

The Family Affluence Scale (FAS-III) [52] is an indicator of young people’s socioeconomic status comprising items on material assets in the family. Scale scores were calculated by summing up the scores of all items. A measure of relative FAS (proportions of low–medium–high in each country) of 20–60–20 percentiles was used in the current study, as has been proposed previously [1].

### 2.4. Statistical Analysis

For data analysis, IBM SPSS Statistics for Windows, Version 20.0 (IBM Corp., Armonk, NY, USA) package was used. The descriptive analyses included means ± standard deviations (SD), as well as percentages. The bivariate associations between categorical variables were conducted using the chi-squared test. Intercorrelations between the variables were calculated using the Spearman rank coefficient. Inferential analyses were based on logistic regression. First, the univariate regression was conducted to check the associations between the analyzed factors and outcomes (school-related well-being and somatic health complaints). In multivariate regression, separate models were built to predict the higher prevalence of somatic complaints. The latter was considered in terms of binary outcomes: the specific symptoms were considered as expressed when they were reported as being experienced more than once a week [1]. Multisite complaints were dichotomized as “high” if at least two of the listed somatic complaints were reported, and “low” if one or none was reported (based on Gobina, 2019 [63]). The putative risk factors included in the multivariate models were—perceived support from teachers, family, and peers, school demand and school satisfaction, and sociodemographic indicators (gender, age group, and family affluence).

The strength of associations was expressed in odds ratios (OR). For inferential analysis, separate support scales were used to calculate an overall social support score (OSSS), reflecting on the number of social support points (teachers, classmates, family) from which a particular adolescent had high social support. This was conducted due to strong associations between different types of support that later in multivariate analyses might have given biased associations due to strong interactions. Since we analyzed three types of support, the OSSS ranged from 0 (none of the supports was high) to 3 (all analyzed social supports were high).

## 3. Results

It was found that, the most common subjective health complaint among respondents was a headache, being expressed among 17.2% of school children. Backache, headache, and stomachache were more common among girls than boys, but statistically significant difference was observed only for backache and stomachache (*p* < 0.001). In most cases, the somatic complaints were expressed more in younger age, though in some cases it was inconsistent (Table 2).

For wider comparisons, additionally, the three analyzed complaints were composed of one indicator—multisite complaint. This score ranged from 0 to 3, which indicated how many complaints a particular student experienced. It was found that multisite complaints were more common among girls than boys (on average, 20.6% and 12.3%; *p* < 0.001) and were associated with age—students aged 13 and 15 years (on average, 17.2% and 19.2%, respectively) reported more complaints than 11 year-olds (on average, 13.1%; *p <* 0.001). The detailed information on the prevalence of the above-mentioned complaints is presented in Table 2.

Social support was measured by considering three types of support—family, classmates, and teachers support (Table 3). High family support was more expressed than the other support types. It can also be noted that high teacher support was usually more prevalent than classmate support. Classmates and teachers support were expressed more among boys, while family support—among girls. Almost all support types had decreasing trends with age.

For further analyses, social support was regarded as a total score composed of classmates, family, and teachers support (as overall social support score—OSSS). It was found that the highest prevalence of full social support (i.e., all three types of social support were high) was among 11 year-olds (42.6% of boys and 41.8% of girls). OSSS was strongly negatively associated with age—in the oldest cohort (15 year-olds) the perceived OSSS prevalence dropped twice (compared with 13 year-olds) in both genders.

Before the multiple regression, the potential predictor variables were checked for possible multicollinearity (Table 4). The correlations were between weak and moderate.

### School-Related Factors and Subjective Somatic Complaints

Analysis of school-related factors and potential outcomes—health complaints—was conducted. For this, the univariate model was calculated first (Table 5). The findings suggested that the strongest school-related factors for perceived somatic complaints were low family and teacher support (except stomachache), low school satisfaction, and the most critical factor was bullying. Bullying odds were highest for all three types of perceived somatic complaints.

In order to specifically see if different types of school-related factors act similarly in predicting the likelihood of multisite complaints, we analyzed them in a multivariate model. At this stage, the multisite complaints were considered as high, if more than one somatic complaint was reported. The analysis showed (Table 6) that it was the male gender and bullying perpetration (OR = 1.7 and OR = 1.8, respectively) that acted most strongly, with a lower relevance of the overall social support (OR = 1.2) and school demand (OR = 1.3).

## 4. Discussion

The main result of this study was that the school-related factors and social support showed consistent associations with multisite somatic complaints in a representative sample of Lithuanian adolescents. The study demonstrated that school-related factors—bullying, school demand, satisfaction, and social support were significant and independent factors for somatic complaints among adolescents.

The similar effect of age and gender was identified in other studies. Previous research has shown that increasing age associates with a higher prevalence of a backache [10,64,65], headache [10,64,65,66,67], and stomach pain [64,65]. In addition, we found that headache and stomach pain were more prevalent among girls than boys. This finding was in line with the previous studies suggesting that the female gender is stronger predictor of different pain symptoms [9,10]; in addition, multisite pain was also more common among girls than specific-site pain [10,12,13]. It is assumed that all biological, cultural, psychological, and social factors (such as pupil dilation, muscle reflexes, differences in cerebral activation or exception, expression, and tolerance of pain, etc.) might contribute to these disparities between males and females regarding the pain responses and management [9,17,19]. Sex hormones play an important role and influence pain sensitivity—pain threshold and pain tolerance in women vary with the stage of the menstrual cycle [17,18]. Puberty also plays an important role—in early stage, the expression of pain complaints is similar among girls and boys, but as puberty progresses, one or more common pain complaints increase more dramatically in girls [9,16]. Psychosocial differences in the perception, expression, and tolerance of pain are likely influenced by a variety of social and psychological processes [9,17,18]. For example, pain catastrophism might explain gender-based differences in the reporting of certain types of pain, with women tending to resort to more hyperbolics [18], or male gender norm dictating increased tolerance of pain among males [68].

Our study results revealed that social support contributed to better somatic outcomes, where high family and teacher support might have acted as strong protective factors against somatic complaints. The importance of family and teacher support for adolescent’s somatic complaints and well-being was widely confirmed in previous studies [29,30,33,46,55,58,65]. Interestingly, our results showed that the type of perceived school-related social support differed between the genders—for boys, support from classmates and teacher was most important, while for girls, family support was the strongest protecting type of support. The prominence of each support source could be based on a variety of cultural, ethical, socialization, or personality factors. During early adolescence, due to biological, psychological, and social role changes, boys are more likely to show problematic behavior that draws attention from teachers, thus, their comprehensive support becomes more important (boys and girls seem to receive differential treatment from teachers) [46]. More specifically, girls might value relational intimacy in a different way or to a different degree than boys, which is possibly why parents’ support becomes more important. Helsen et al. (2000) noted that shifts from family support to peer support occur more gradually in boys than girls, which means that relationships with classmates become more and more essential for boys as they mature [69].

Our study found a consistent relationship of somatic complaints with bullying. Bullying perpetration was mostly associated with all types of subjective somatic complaints in all age and gender groups. This pattern was found in other studies, where bullying perpetration is considered as the most essential factor for adolescent somatic outcomes [5,38,42]. Furthermore, these studies showed that bullying has serious long-term effects on health and well-being later in life—childhood psychiatric disorders (including conduct disorder, hyperactivity disorder, attention deficit, or oppositional defiant disorder) [42], backache, headache, fatigue, abdominal pain, dizziness, and sleep problems [5,41,70]. Involvement with bullying in any role is a very strong predictor of negative health, risk behavior (smoking, drinking, drug usage, long-term illness, depression, psychiatric problems, etc.) [42,71], and social outcomes (illegal behaviors) in later adulthood [42,70].

To conclude, our study indicated that somatization was affected by quite a wide range of school-related factors, where all types of school contents (bullying, school-related demand and support, and school satisfaction) played similarly important roles for the school children’s somatic outcomes. Adolescents with multisite pain were more likely to report impaired quality of life [12,72] and higher levels of other health complaints like anxiety and depression [12,73], which could further negatively affect the psychosocial development and daily functioning over time [14,73,74]. This study extended the limited research in this area by analyzing not only a possible predictor of the expected outcomes, but also by reporting the likelihood of significant somatic complaints associated with school-related risk factors, as a complex.

When talking about the limitations of this study, it should be noted that self-reported somatic complaints might not always reflect the actual prevalence of this problem due to other important external factors—emotional background of adolescents during the data collection, different threshold of experienced health complaints, possibly blurred distinctions between health and illness, etc. In this study, we used the overall school-related social support in order to evaluate the total support in the learning environment of school children. This measure ranged from 0 to 3 and was limited in that it implied the same weight for all three types of support—teachers, peers, and parents, which might not be necessarily equal in their relevance. On the other hand, our data were based on a rich dataset that enabled the assessment of different aspects, not only of social support but of psychosomatic symptoms as well. Additionally, it should be noted that in our study, bullying perpetration was measured through questions, which were preceded by the definition of bullying [59], but such aggression behavior experiences that were outside the scope of the bullying definition could also have been harmful [75]. Therefore, it should be taken under consideration that HBSC bullying perpetration definition has not yet been validated as a direct measure of bullying, despite its wide usage in empirical studies across multiple countries. When talking about data analysis, multilevel modeling was not used, and it could also be considered as a limitation.

## 5. Conclusions

With a few exceptions, the majority of the study results confirmed the findings of other researchers. Most common subjective health complaints among respondents was a headache. Backache, headache, and stomachache were more prevalent among girls than boys. Multisite complaints were more common among girls and were associated with age—older ones reported more complaints. School-related factors (bullying, school satisfaction, and demand) and social support showed consistent associations with multisite somatic complaints among adolescents. High social support contributed to better somatic outcomes. Thus, high family and teacher support were found to be multidimensional and to act as a possible protective factor against somatic complaints. Additionally, bullying perpetration was mostly associated with all types of subjective somatic complaints in all age and gender groups. This provides an important information that could be used in both clinical and public health practices to maximize the management and prevention of somatic complaints among adolescents. Furthermore, somatic health complaints in adolescents should be managed in a multidisciplinary manner, in particular taking into account the possible buffering factors which co-exist in a school-related environment.

## Figures and Tables

**Table 1:** Study sample by age, gender, school grade, and family affluence.

Variable	*n*	%
Gender		
Boys	2910	50.8
Girls	2820	49.2
Age group		
11-year old	2015	35.2
13-year old	2017	35.2
15-year old	1698	29.6
School grade (class)		
5th grade	2057	35.9
7th grade	2000	34.9
9th grade	1673	29.2
Family affluence		
Low (0–5 pts)	1317	23.8
Medium (6–9 pts)	3125	56.4
High (10–12 pts)	1101	19.9

**Table 2:** Prevalence of subjective health complaints by age and gender (in percentage).

Characteristic	Total	Boys			Girls		
		11 Years	13 Years	15 Years	11 Years	13 Years	15 Years
Backache							
Rarely or Never	89.0	91.6	90.0	87.5	92.3	87.9	84.5
More than Once a Week	11.0	8.4	10.0	12.5	7.7	12.1	15.5
Headache							
Rarely or Never	82.8	87.5	87.0	90.0	83.5	76.9	71.7
More than Once a Week	17.2	12.5	13.0	10.0	16.5	23.1	28.3
Stomachache							
Rarely or Never	88.5	89.8	92.0	82.5	88.0	85.6	83.0
More than Once a Week	11.5	10.2	8.0	7.5	12.0	14.4	17.0
Multisite Complaints							
0 symptoms	62.1	70.7	69.4	67.6	66.7	53.7	44.5
1 symptom	21.4	17.9	17.7	19.6	18.5	24.8	30.0
2 symptoms	10.4	6.9	7.9	7.3	10.5	14.0	16.0
3 symptoms	6.1	4.5	4.9	5.5	4.3	7.5	9.6

**Table 3:** Prevalence of perceived social support by age and gender.

Characteristic	Total	Boys			Girls		
		11 Years	13 Years	15 Years	11 Years	13 Years	15 Years
Family Support							
High	68.4	77.8	67.5	57.2	80.5	69.2	58.3
Average or Low	31.6	22.2	32.5	42.8	19.5	30.8	41.7
Classmates Support							
High	47.5	59.9	52.7	47.8	53.6	36.7	34.0
Average or Low	52.5	40.1	47.3	52.2	46.4	63.3	66.0
Teachers Support							
High	53.3	65.2	48.5	58.8	67.6	43.2	36.2
Average or Low	46.7	34.8	51.5	41.2	32.4	56.8	63.8
Overall social support score							
0 pts	17.4	11.0	15.8	22.7	9.7	19.7	25.4
1 pts	27.2	18.1	29.1	29.3	19.9	31.8	35.0
2 pts	27.5	28.4	26.6	27.1	28.6	28.4	25.7
3 pts	28.0	42.6	28.5	20.8	41.8	20.2	13.9

**Table 4:** Inter-correlations and descriptive statistics of somatic complaints and social support.

Measure	Mean	SD	1	2	3	4	5	6
1. Headache	1.17	0.375	1.00	0.39 *	0.30 *	−0.06 *	−0.04	−0.04
2. Stomachache	1.11	0.316		1.00	0.34 *	−0.06 *	−0.01	−0.01
3. Backache	1.11	0.311			1.00	−0.08 *	−0.05 *	−0.06 *
4. High Parents Support	0.69	0.463				1.00	0.22 *	0.29 *
5. High Peers Support	0.48	0.500					1.00	0.36 *
6. High Teachers Support	0.51	0.500						1.00

Note: * *p* < 0.05.

**Table 5:** School factors and somatic complaints: univariate logistic regression.

Characteristic	Backache		Headache		Stomachache	
	Odds Ratio	CI 95%	Odds Ratio	CI 95%	Odds Ratio	CI 95%
Family Support						
High	1.00		1.00		1.00	
Average or Low	1.73	1.46–2.06	1.42	1.23–1.65	1.50	1.27–1.79
Classmates Support						
High	1.00		1.00		1.00	
Average or Low	1.38	1.16–1.64	1.21	1.06–1.40	1.07	0.91–1.27
Teachers Support						
High	1.00		1.00		1.00	
Average or Low	1.52	1.28–1.81	1.21	1.05–1.40	1.04	0.90–1.23
Been Bullied						
No	1.00		1.00		1.00	
Yes	1.63	1.37–1.95	1.47	1.28–1.70	1.67	1.40–1.99
School Demand						
A little or Not at all	1.00		1.00		1.00	
A lot	1.31	1.11–1.56	1.32	1.15–1.52	1.15	0.97–1.34
School Satisfaction						
Like a lot	1.00		1.00		1.00	
Not at all	1.52	1.25–1.85	1.50	1.27–1.77	1.33	1.10–1.63

**Table 6:** Multisite complaints and associated factors: logistic regression model.

Characteristic	Model 1 *	Model 2 **
	Odds Ratio (CI 95%)	Odds Ratio (CI 95%)
Been Bullied		
Yes	1.89 (1.63–2.20)	1.78 (1.51–2.08)
School Demand		
A lot	1.50 (1.30–1.73)	1.34 (1.15–1.56)
School Satisfaction		
Not at all	1.52 (1.29–1.80)	1.25 (1.04–1.50)
Age		
11-year old	1.37 (1.15–1.63)	1.24 (1.03–1.50)
13-year old	1.53 (1.28–1.83)	1.42 (1.16–1.73)
Gender		
Girls	1.80 (1.56–2.08)	1.87 (1.60–2.18)
Family affluence		
Low	1.56 (1.25–1.93)	1.37 (1.09–1.73)
Medium	1.04 (0.85–1.26)	1.02 (0.83–1.25)
Overall social support		
Not sufficient	1.58 (1.37–1.82)	1.21 (1.03–1.42)

* Model 1—univariate regression. ** Model 2—multivariate regression, adjusted for all variables in the model.
